# Short-term outcomes of treatment switch to faricimab in patients with aflibercept-resistant neovascular age-related macular degeneration

**DOI:** 10.1007/s00417-024-06421-0

**Published:** 2024-02-28

**Authors:** Miklos Schneider, Jakob Bjerager, Delila Hodzic-Hadzibegovic, Oliver Niels Klefter, Yousif Subhi, Javad Hajari

**Affiliations:** 1 Department of Ophthalmology, Rigshospitalet, Valdemar Hansens Vej 1-23, 2600 Glostrup, Denmark; 2 Department of Ophthalmology, Semmelweis University, Budapest, Hungary; 3 Department of Clinical Medicine, University of Copenhagen, Copenhagen, Denmark; 4 Department of Clinical Research, University of Southern Denmark, Odense, Denmark

**Keywords:** Age-related macular degeneration, AMD, Faricimab, Aflibercept, Intravitreal injection, Anti-VEGF, Neovascular

## Abstract

**Purpose:**

To report short-term outcomes of treatment switch to faricimab in real-world patients with aflibercept-resistant neovascular age-related macular degeneration (AMD).

**Methods:**

Single-center, retrospective cohort study with chart-review using electronic injection database, electronic medical records, and optical coherence tomography (OCT) data from May to September 2023.

**Results:**

A total of 50 eyes of 46 patients were analyzed. Faricimab treatment led to absence of fluid in 32% of the eyes and a reduction of fluid in 84% of the eyes. There was a statistically significant decrease in central retinal thickness (CRT) and pigment epithelial detachment (PED) height in those that responded to the switch (median difference: − 31 μm, IQR: 55, *p* < 0.0001 and median difference: − 21 μm, IQR: 36, *p* < 0.0001, respectively) and a statistically significant increase in CRT (median difference: + 19 μm, IQR: 20, *p* = 0.0143) and no change in PED height (median difference: + 22 μm, IQR: 64, *p* = 0.1508) in those that did not.

Best-corrected visual acuity (BCVA) showed marginal decrease with low statistical significance. No ocular or systemic safety events were observed.

**Conclusions:**

Our findings suggest that switching to faricimab is generally safe and effective in patients with neovascular AMD who are otherwise difficult to treat and have residual fluid despite frequent injections with aflibercept. We observed a high rate of morphological response to the treatment switch, improvement of anatomical parameters with about one-third of patients having dry macula following a single injection, and a marginal change in BCVA. Sustainability of these results requires further investigation.

**Study registration:**

ClinicalTrials.gov registration number: NCT06124677. Date of registration: 09/11/2023, retrospectively registered.

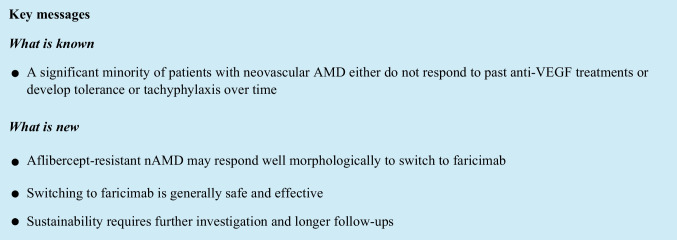

**Supplementary Information:**

The online version contains supplementary material available at 10.1007/s00417-024-06421-0.

## Introduction

Neovascular age-related macular degeneration (AMD) is a prevalent and debilitating retinal disease that poses a significant public health concern. It is the leading cause of irreversible vision loss in the developed countries among individuals aged 50 and older, impacting millions worldwide. The incidence of the disease is expected to increase [1, 2], which has placed a growing burden on both patients and healthcare systems worldwide. The introduction of intravitreal anti-vascular endothelial growth factor (anti-VEGF) therapies has transformed the management of neovascular AMD, providing hope for patients by significantly slowing disease progression and preserving vision [3]. However, despite their success, these therapies are not without limitations, including issues with non-responsiveness, tachyphylaxis, and the necessity for frequent intravitreal injections [4, 5]. A subset of patients experiences limited or diminishing therapeutic effects over time, leading to increased treatment burden and reduced quality of life [6]. Additionally, with the increasing number of patients impacted by neovascular AMD, the need for intravitreal anti-VEGF treatments is also predicted to rise in the near future [2], which is expected to be a significant challenge for every healthcare system. The latest projection predicts a 50% increase by 2027 in Denmark [7].

Faricimab (Vabysmo®, Roche/Genentech, Basel, Switzerland) was recently approved and introduced as another anti-VEGF agent for the treatment of neovascular AMD [8, 9]. Faricimab targets both the vascular endothelial growth factor (VEGF) and angiopoietin-2 (Ang-2) pathways, and its dual pharmacodynamic properties potentially allow for a more lasting effect and lower treatment burden [10].

The phase 3 faricimab clinical trials (TENAYA and LUCERNE) demonstrated non-inferiority compared to aflibercept, but with longer treatment intervals with approximately 80% of patients being on a 12-week or 16-week dosing schedule at 48 weeks [11]. Ocular adverse events observed in both trials were in line with what is typically seen in patients undergoing intravitreal treatment, and the occurrence rates were similar in both the faricimab and aflibercept groups [11].

According to the Danish national recommendations and our local guidelines, patients with neovascular AMD have routinely been started on aflibercept treatment with an observe-and-plan regimen. According to this strategy, treatment intervals can be shortened to 4 weeks for cases with persisting fluid. However, even with such intensive treatment, a substantial number of cases can be treatment resistant. Such cases receive injections every 4 weeks, which leads to a considerable treatment burden for the patient. Therefore, we explored the benefits of faricimab in these cases.

The purpose of this study is to report the short-term outcomes of switching treatment to faricimab in real-world patients with aflibercept-resistant neovascular AMD.

## Methods

### Ethical approval and consent

This retrospective single-center chart review was conducted at the Department of Ophthalmology, Rigshospitalet, Glostrup, Denmark, a large regional highly specialized tertiary center.

The research and data handling were conducted in compliance with local laws, the tenets of the Declaration of Helsinki and its amendments. Based on the decision of the Regional Ethics Committee of the Capital Region of Denmark, the study protocol was exempt from institutional review board approval, as the research involved retrospective data collection from routine clinical procedures within indication (case nr.: F-23059408, date of decision: 01/11/2023). The Research Legal Department of the Capital Region of Denmark has approved the patient confidentiality and data flow protocol of the project (approval nr.: p-2023–14984, date of decision: 03/11/2023). The study protocol was registered at ClinicalTrials.gov, registration number: NCT06124677.

### Patient selection and inclusion and exclusion criteria

In this study, we reviewed patients who received intravitreal faricimab injections between May 24 and September 4, in 2023. These patients were switched directly to faricimab treatment from aflibercept without a new loading phase. The date of the first faricimab injection was defined as baseline.

Inclusion criteria were 50 years of age or older, presence of neovascular AMD, previous intravitreal anti-VEGF treatments with a minimum of 6 monthly aflibercept (Eylea®, Bayer, Leverkusen, Germany) injections in the study eye, and persisting intraretinal fluid (IRF) or subretinal fluid (SRF) or both on OCT scans at 4 weeks following the last aflibercept injection, despite at least 3 consecutive monthly aflibercept injections before switching to faricimab. Ranibizumab (Lucentis®, Novartis, Basel, Switzerland) injections were permitted during the treatment, provided the patients were switched back to aflibercept and had received at least 3 consecutive monthly aflibercept injections before switching to faricimab (Supplementary Fig. 1.)

Exclusion criteria were neovascular conditions other than AMD (e.g., choroidal neovascularization [CNV] secondary to other causes) or co-existence of other retinal disease in the study eye, significant optical media opacities that would result in poor imaging quality on optical coherence tomography (OCT) scans in the study eye, or intraocular surgery in the study eye 3 months before or within 1 month after the treatment switch. No further restrictions were enforced.

Examinations and treatments were carried out under real-world routine clinical care conditions, according to in-house standards and local guidelines.

### Procedure

During the selected time period, patients received intravitreal faricimab injections given by trained injection nurses or ophthalmology residents. The procedures followed local standard protocol, that included the use of 2–3 drops of topical tetracaine anesthesia, use of an eye speculum, topical 5% povidone–iodine disinfection, 30G-needle, injection site 3.5-mm posterior from the limbus marked by calipers in the supertemporal or the inferotemporal quadrant, sterile cotton tip tamponade at the site of injection after removal of the needle, and no post-procedure antibiotics [12].

Follow-up check-ups were scheduled for 4 weeks following injections. In case of dry macula at 4 weeks following the faricimab injection, additional weekly check-ups were scheduled to determine durability of the treatment, after which a new treatment interval was set up in case the fluid reappeared (Supplementary Fig. 1.)

### Data collection

The data collection was carried out by using the center’s internal injection and imaging database and by reviewing patient charts. Collected data included demographics (age, sex), treatment history (number and type of previous treatments), best corrected visual acuity (BCVA), central retinal subfield thickness (CRT), presence of pigment epithelial detachment (PED) and PED height if applicable, type of retinal fluid (intra- or subretinal or both) before the treatment switch, presence or absence of residual fluid in case of favorable therapeutic response, time of reappearance of the fluid in case of dry macula after the first faricimab injection, and adverse events after the treatment switch. Visual acuity was measured in Snellen and was subsequently converted to Early Treatment Diabetic Retinopathy Study (ETDRS) BCVA score with the “ETDRS = 85 + 50 × log_10_ (Snellen Fraction)” formula [13] for statistical calculations. CRT and PED measurements and retinal fluid type classifications were done using IMAGEnet 6 (software version 1.31.18920, Topcon Corp, Tokyo, Japan) by evaluating OCT scans acquired by the DRI OCT-1 Triton swept-source OCT device (Topcon Corp, Tokyo, Japan). CRT values were determined automatically by the software, correct placement of the ETDRS grid and accuracy of boundary detection was checked by two independent observers (MS, DHH), and segmentation was corrected manually in case of boundary detection artifacts. PED height measurement was performed by visually locating the highest point of the PED within a 3-mm circle around the fovea (corresponding to the central and inner sectors on the ETDRS grid), and manually measuring the distance between the elevated retinal pigment epithelium (RPE) and the Bruch’s membrane (BM), using the software’s electronic caliper perpendicular to the BM. When it was doubtful where the highest point was, several measurements were taken in the suspected areas, and the highest value was selected for the analysis. Each measurement was taken independently by two observers (MS, DHH); discrepancies were resolved by discussion.

### Outcome measures

The primary endpoint of the study was the proportion of patients responding morphologically to the treatment switch, defined as reduction or disappearance of the intra- or subretinal fluid on OCT scans at 4 weeks following a single injection of faricimab (responders). Patients showing no change or increase in the amount of fluid on OCT scans following the treatment switch were defined as non-responders.

Secondary endpoints were durability of a single injection in cases of optimal response without residual fluid (i.e., time of reappearance of the fluid), differences in patient characteristics between individuals responding and not responding to faricimab, changes in BCVA after the treatment switch, and changes in CRT and PED height after the treatment switch. Safety endpoints were frequency of ocular (including, but not limited to endophthalmitis, occlusive vasculitis, sterile intraocular reaction, retinal brakes, retinal detachment, intraocular bleeding, intraocular pressure increase) and systemic adverse events (including, but not limited to stroke, acute myocardial infraction, other cardiovascular events, or death).

### Statistical analysis

Statistical analyses were conducted using RStudio v. 2022.7.1.554 (RStudio Team (2022). RStudio: Integrated Development Environment for R. RStudio, PBC, Boston, MA, USA. URL http://www.rstudio.com/) with R version 4.3.1 (R Core Team (2022) R: A language and environment for statistical computing. R Foundation for Statistical Computing, Vienna, Austria. URL https://www.R-project.org/) and GraphPad Prism v9.4.1681 for Windows (GraphPad Software, Boston, MA, USA). Shapiro–Wilk test was used to test for normality. Normally distributed parameters were reported with mean and standard deviation, whereas median and interquartile range were reported for non-parametric data.

To test for true differences between groups of unpaired observations, two-sided non-paired Student’s *t* test was used for parametric data and non-paired, two-sided Wilcoxon rank test was used for non-parametric data. For paired observations, two-sided paired Student’s *t* test and two-sided, paired Wilcoxon rank test were used, respectively. For Wilcoxon rank tests, approximate *p* values were used. Fischer’s exact test was used to compare categorical variables. *P* values of < 0.05 were considered statistically significant.

## Results

### Demographics, prior injections, and fluid type

Our injection database search identified 73 eyes that received faricimab treatments in the selected time frame. Twenty-three eyes needed to be excluded from the analysis due to either being switched from ranibizumab (*n* = 13), having the wrong diagnosis (*n* = 3), not having fluid before the treatment switch (*n* = 4), due to undergoing cataract surgery shortly after the treatment switch (*n* = 1), or missing 4-week data (*n* = 2). In total, 50 eyes of 46 patients were included in the analysis.

The average age of the included patients was 76.4 years (range: 54–89 years), and there were 46% females (*n* = 21) and 54% males (*n* = 25).

Prior to the treatment switch to faricimab, all eyes (*n* = 50) received aflibercept with a median of 33 injections per eye (IQR: 26, range: 6–111), and 42% of the eyes (*n* = 21) received additional ranibizumab injections with a median of 6 injections per eye (IQR: 4, range: 1–47).

According to the inclusion criteria, all included eyes had persisting fluid with 78% (*n* = 39) having SRF, 12% (*n* = 6) having IRF, and 10% (*n* = 5) having both SRF and IRF. All patients demonstrated presence of PED.

### Efficacy

**Fig. 1:**
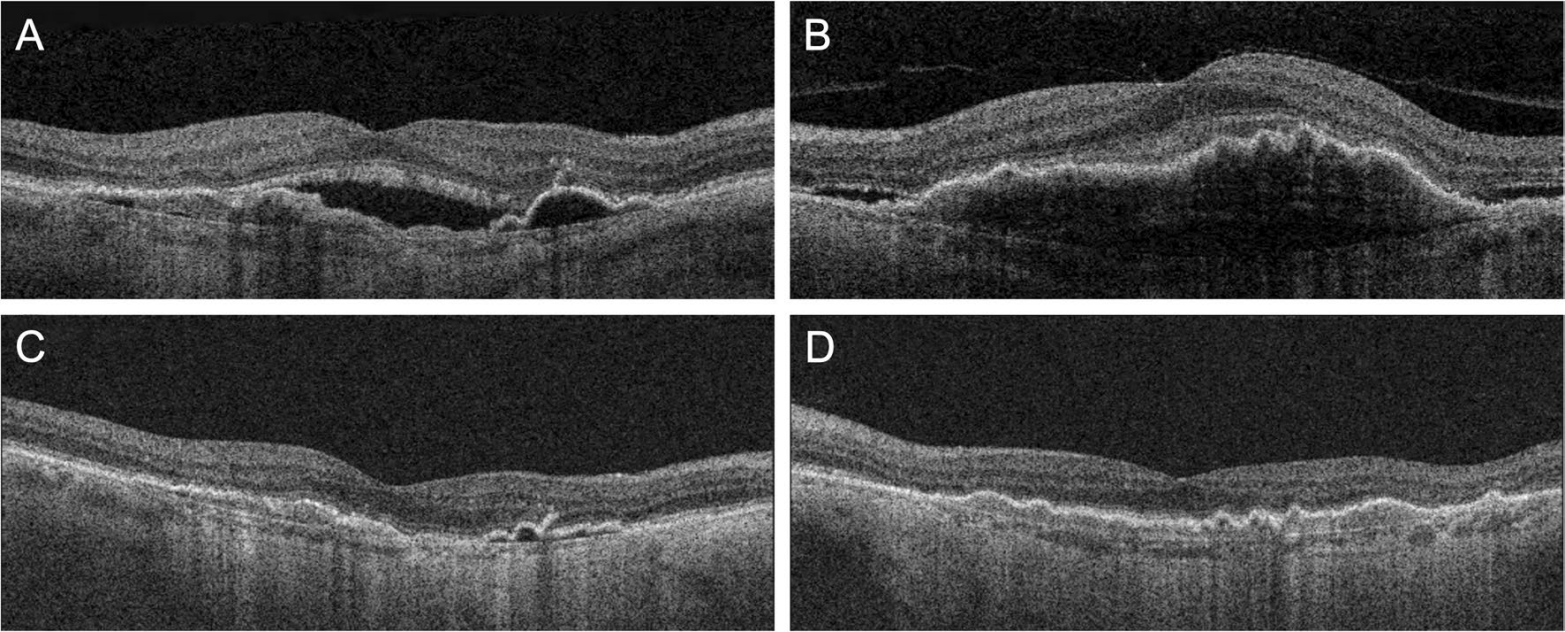
Representative case for optimal response on both eyes. **A**, **B** Optical coherence tomography scans of the right and left eye at baseline; **C**, **D** scans of the right and left eye at follow-up. The patient is an 82-year-old female, who previously received 57 and 18 aflibercept injections in the right and left eye, respectively. Treatment in both eyes was switched to faricimab and the patient responded optimally to a single injection with dry macula bilaterally at 4 weeks. Additionally, there was a significant reduction in the height of the pigment epithelium detachments in both eyes at 4 weeks. Best-corrected visual acuity was 20/20 at baseline in both eyes. At follow-up, vision in the right eye worsened to 20/28 and improved to 20/20 at subsequent visits. Left eye maintained 20/20 at follow-up and subsequent visits

Following the treatment switch to faricimab at week 4, the macula was completely dry in 32% of the eyes (*n* = 16), and a reduction of the fluid and residual edema was seen in an additional 52% of eyes (*n* = 26), which resulted in a total response rate of 84% (*n* = 42). Durability of the treatment (i.e., time to reappearance of the fluid) among the optimally responding subgroup had a median of 6 weeks (IQR: 1, range: 5–9). Figure 1 shows OCT scans of a representative patient from our cohort who received faricimab in both eyes simultaneously and responded optimally with dry macula after the switch on both eyes. A smaller proportion, 16% (*n* = 8) of eyes did not respond to the switch. There were no statistically significant differences between the responder- and the non-responder groups in age, sex, previous injection count or baseline BCVA. Detailed tabulation of the demographics of the responder and non-responder groups can be found in Table 1.

**Table 1:** Demographics of responders and non-responders after one intravitreal injection with faricimab

	Responders	Non-responders	*p* value
Number (% of total)	42 (84.0%)	8 (16.0%)	-
Age, years—median (IQR)	79.5 (11.75)	76.5 (6)	0.4343
Females—number (%)	19 (45.2%)	3 (37.5%)	1.00
Number of previous aflibercept injections—median (IQR)	33 (26.25)	45 (23.25)	0.6147
Number of previous ranibizumab injections—median (IQR)	0 (3)	4.5 (6)	0.1693
Total number of previous injections count—median (IQR)	33 (25.5)	50 (24)	0.4745
BCVA prior to switch, EDTRS letters—median (IQR)	74 (15)	71 (19)	0.3006

*BCVA* Best-corrected visual acuity; *EDTRS* Early Treatment Diabetic Retinopathy Study; *IQR* inter-quantile range

All patients were switched from aflibercept to faricimab. Responders were defined by the absence or reduction of intra- or subretinal fluid 4 weeks after the first intravitreal injection of faricimab. Non-responders had unchanged or increased amounts of fluid

Grouping the eyes by fluid type at baseline, we found a response rate of 82.05% (*n* = 32) among those with SRF (*n* = 39) with 33.3% (*n* = 13) of them having dry macula at 4 weeks. All eyes that had IRF only (*n* = 6) responded to the switch and half of them (*n* = 3) had dry macula at the follow-up. Eyes having both SRF and IRF (*n* = 5) had an 80% response rate (*n* = 4), and none of them had dry macula at follow-up.

**Fig. 2:**
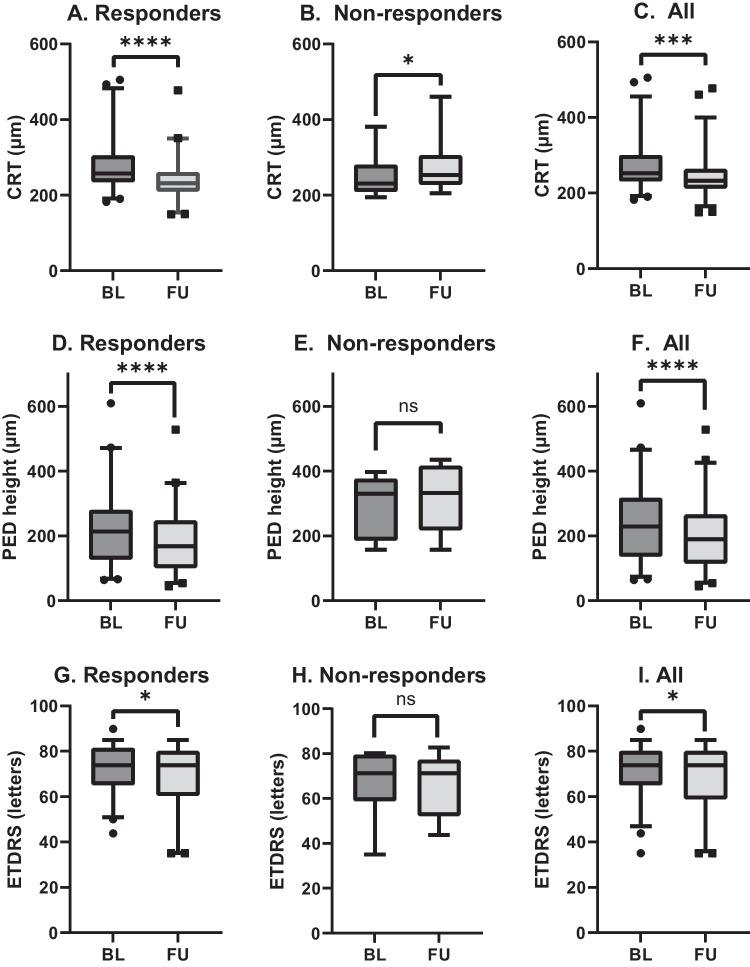
Box plots with 5–95% ranges comparing values at baseline and 4-week follow-up. **A**–**C** Central retinal thickness (CRT) for responders, non-responders, and the total study population, respectively. **D**–**F** Pigment epithelium detachment (PED) height for responders, non-responders, and the total study population, respectively. **G**–**I** Best-corrected visual acuity in Early Treatment Diabetic Retinopathy Study (ETDRS) letters for responders, non-responders, and the total study population, respectively. BL: baseline; FU: follow-up; ns: *p* value statistically non-significant; *p value <0.05; ***p value < 0.001; ****p value < 0.0001

There was a highly statistically significant decrease in CRT in the responder group 4 weeks after the switch (median difference: − 31 μm, IQR: 55, range: [ −]154–39, *p* < 0.0001) and a statistically significant increase in CRT in the non-responder group (median difference: + 19 μm, IQR: 20, range: 10–79, *p* = 0.0143) (Fig. 2a–c). Similarly to the CRT, PED height also showed a highly significant decrease in the responder group (median difference: − 21 μm, IQR: 36, range: [ −]307–2, *p* < 0.0001); while we observed no changes in PED height in the non-responder group (median difference: + 22 μm, IQR: 64, range: [ −]38–91, *p* = 0.1508) (Fig. 2d–f).

We found no statistically significant change in BCVA at 4 weeks after the switch in the non-responder group (*p* = 0.9163). However, a marginal decrease in BCVA with low statistical significance was seen when analyzing the responder group and the total study population (with *p* values of 0.0207 and 0.0292, respectively) (Fig. 2g–i).

Detailed tabulation of the CRT, PED height and BCVA data can be found in Table 2.

**Table 2:** Central retinal thickness, pigment epithelial detachment height and best-corrected visual acuity before and after the switch from aflibercept to faricimab

	Responders (*n* = 42)			Non-responders (*n* = 8)			All (*n* = 50)		
	Baseline	Follow-up	*p* value	Baseline	Follow-up	*p* value	Baseline	Follow-up	*p* value
CRT, µm, median (IQR)	257 (67)	232 (48)	< 0.0001	231 (36)	254 (65)	0.0143	252 (66)	232 (49)	0.0001
PED height, µm, median (IQR)	213 (147)	168 (139)	< 0.0001	330 (136)	333 (181)	0.1508	229 (173)	189 (145)	< 0.0001
BCVA, ETDRS letters, median (IQR)	74 (15)	74 (17)	0.0207	71 (19)	71 (21)	0.9163	74 (15)	74 (21)	0.0292

*BCVA* Best-corrected visual acuity; *CRT* central retinal thickness; *EDTRS* Early Treatment Diabetic Retinopathy Study; *IQR* inter-quantile range; *PED* pigment epithelial detachment

All patients were switched from aflibercept to faricimab. Responders were defined by the absence or reduction of intra- or subretinal fluid 4 weeks after the first intravitreal injection of faricimab. Non-responders had unchanged or increased amounts of fluid

**Fig. 3:**
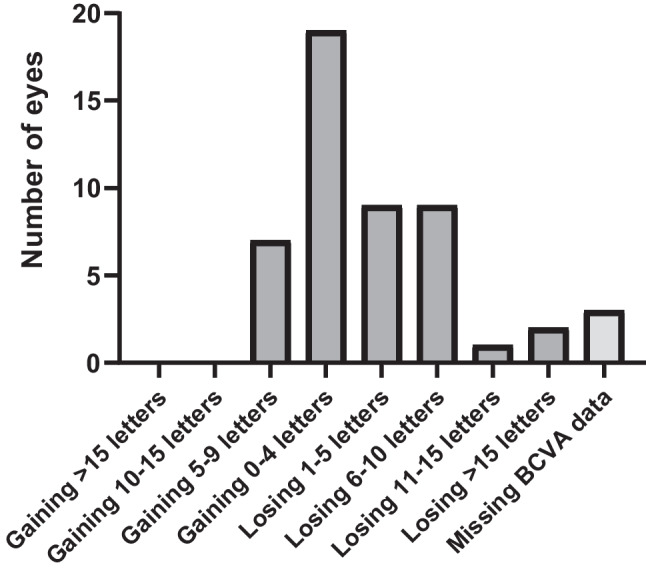
Distribution of eyes by best-corrected visual acuity change. Changes are reported in Early Treatment of Diabetic Retinopathy Study (ETDRS) letters. BCVA: best-corrected visual acuity

The majority (90%, *n* = 45) of the eyes had stable visual acuity (i.e., no clinically relevant changes, BCVA score within ± 15 ETDRS letters [14]) at follow-up. Most eyes (52%, *n* = 26) maintained the same BCVA ETDRS score or gained letters, 18% (*n* = 9) lost 1–5 letters, 24% (*n* = 12) lost more than 5 letters, and 6% (*n* = 3) lost more than 10 letters. No eyes gained more than 9 letters, and 2 eyes (4%) lost more than 15 letters. These latter two patients experienced visual acuity worsening of 20 and 30 letters, respectively, despite favorable morphological response after the treatment switch. No evidence of RPE rupture, subretinal hemorrhage, occlusive vasculitis, or other identifiable morphological causes or potentially contributing co-morbidity were found in these cases, and their vision did not change significantly over time at subsequent check-ups. Three eyes had missing data regarding change in BCVA. Figure 3 shows the distribution of eyes by BCVA change in detail, in 5-letter increments.

### Safety

During the selected time frame, no endophthalmitis, occlusive vasculitis, sterile intraocular reaction or other ocular adverse event were seen, and no systemic adverse events were noted following the treatment switch.

## Discussion

Our study focused on the anatomical and visual outcomes of a single injection of faricimab in patients having residual fluid despite frequent aflibercept treatments and found a high rate of morphological response to the treatment switch. Currently, there is an unmet need for treatment options for a significant minority of patients with neovascular AMD who either do not respond to past anti-VEGF treatments or develop tolerance or tachyphylaxis [5] over time. Patients who develop tachyphylaxis may respond well to a therapy switch, sometimes similarly to our study, with a very high success rate [4]. Moreover, switch patients are typically not included in clinical trials; therefore, real-world data on these patient groups is highly relevant for retinal physicians.

Following the pivotal TENAYA and LUCERNE trials [11] and the approval of faricimab in the USA [8] and the European Union [9], several authors reported on their initial experiences with the drug. A handful of real-world studies reported on short-term results [15–24], but due to the novelty of the drug, studies with 6-months of follow-up [25, 26] or longer [27] are only available in a very limited number.

According to our knowledge, this is the first real-world report on faricimab use from the Nordic countries.

Some of the recent papers similarly to our study, report on results with neovascular AMD patients who were switched over to faricimab due to treatment resistance with previous anti-VEGF agents [15–18, 21, 23, 25, 27]. Most of these studies agree that switching over to faricimab proved to be an effective treatment for neovascular AMD for anatomic changes [15–17, 21, 23], with preserved [15, 17, 23] or improved visual outcomes [18, 21], with longer [17, 18, 25, 27] or similar treatment intervals [23] compared to prior anti-VEGF therapy, and is considered safe [16].

Similarly to our research, Inoda et al. [16] aimed to assess the effect of a single dose of faricimab in patients with prior anti-VEGF history. They described no significant change in central retinal thickness or BCVA following the switch. However, they observed a potential decrease in central choroidal thickness and suggested that faricimab is generally safe and may improve the choroidal circulation. It needs to be mentioned that their cohort consisted of well-controlled patients in their maintenance phase of their treatment, which may explain their results [16] being inconsistent with previous studies reporting both anatomical and visual improvement [21, 22, 26].

Our study found significant decrease in both CRT and PED height following the switch, and a borderline decrease in BCVA with low significance. There was a high rate of some level of response to the treatment switch with about one-third of eyes having completely dry macula after a single injection. Our results are similar to those in the TRUCKEE study, that examined both switch and naïve patients, where authors found complete resolution of the IRF and SRF in 12.3% and 37.2% respectively at the first check-up following a single injection of faricimab in the subgroup that was switched from aflibercept [26].

There is a general agreement among retina specialists that persisting intraretinal fluid indicates a worse prognosis in neovascular AMD and is associated with worse visual and anatomical outcomes compared to subretinal fluid [28]. However, there seems to be no consensus on how much subretinal fluid is tolerable. The FLUID study has shown that patients with neovascular AMD who tolerated some SRF achieved comparable visual acuity outcomes with fewer injections compared to those whose treatment aimed to resolve all SRF completely [29]. Some studies suggest that SRF is compatible with good visual and anatomical outcomes [28], and refractory SRF may still allow for maintained or improved vision, even if located subfoveally [30, 31]. However, another study by Ehlers et al. indicated that the high volatility of subretinal fluid can lead to worse visual and anatomical outcomes [32].

There is an ongoing debate on whether having a small residual subretinal fluid (SRF) during anti-VEGF treatment in neovascular AMD has a protective effect or not. A post-hoc analysis of the CATT trial showed that eyes with foveal SRF had better visual acuity at the 5-year mark compared to eyes without SRF. This effect was particularly pronounced when compared to the 2-year mark. The authors point at several hypotheses, such as the SRF serving to protect the photoreceptors from potential toxicity related to direct contact with the underlying diseased RPE, or providing trophic support to the overlying retina, or protecting the photoreceptors from direct infiltrative damage by serving as a fluid buffer between the outer segments and the CNV below it, or the SRF itself containing neuroprotective substances [30]. These findings indicate that the requirement to administer monthly injections for the management of refractory SRF could be questioned. However, it is important to note that the evidence on this topic is limited, and further investigations are needed to fully understand the impact of residual SRF during anti-VEGF treatment in neovascular AMD.

The majority of our study participants had SRF, and we observed a high rate of favorable morphological response in our cohort. None of our patients experienced significant visual acuity gain from the switch, which was expected, since these patients were in the maintenance phase of their treatment after many anti-VEGF injections. However, it should be noted that despite most of our patients had stable visual acuity after the treatment switch, the cohort showed a marginal decrease in BCVA, with almost a quarter of the participants losing more than 5 letters, and two patients having experienced significant vision loss after the treatment switch, despite good anatomic response. These findings should be interpreted in context with the highly selected population of subjects included in our study that had long-standing disease not responding to previous treatments or needing frequent injections. Disease chronicity might cause a higher susceptibility to the worsening of photoreceptor function than in a general population of AMD-patients assessed at any given period. Nevertheless, our findings may serve as a warning sign that aggressive drying in certain patients with neovascular AMD and persistent SRF under chronic anti-VEGF treatment might paradoxically lead to worse visual outcomes in some cases, supporting the theory of SRF having a potential protective effect.

Limitations to our study should be acknowledged. Although a strength of this study is that we report real-life evidence and outcomes according to the prevalence distribution of various fluids, one important limitation is that our study was not sampled specifically to find differences between fluid types. Therefore, comparisons between fluid types should be interpreted with caution. Additionally, our study focused on outcomes after a single faricimab injection only. Whether this high response rate to the treatment switch is sustainable in the long term would require longer follow-ups.

To conclude, in this study, we observed a high rate of morphological response to treatment switch from intravitreal aflibercept to faricimab with about one-third of patients showing completely dry macula following a single injection. There was a significant reduction in CRT and PED height and a marginal decrease in BCVA. Our findings suggest that switching to faricimab is generally safe and effective in patients with neovascular AMD who have residual fluid despite frequent injections. Sustainability of these results requires further investigation and longer follow-ups.

### Supplementary Information

Below is the link to the electronic supplementary material.

Supplementary file1 (PDF 83 KB)

Supplementary file2 (DOCX 12 KB)

## Data Availability

The datasets generated and/or analyzed during the current study are available from the corresponding author on reasonable request.
