# Supplementary figures and images for: Short-term outcomes of treatment switch to faricimab in patients with aflibercept-resistant neovascular age-related macular degeneration
Source: Graefes Arch Clin Exp Ophthalmol. 2024 Feb 28;262(7):2153–62. doi: 10.1007/s00417-024-06421-0 (PMC11222265; doi:10.1007/s00417-024-06421-0)

## PAST TREATMENTS

## SWITCH

## BASELINE

## FOLLOW-UP

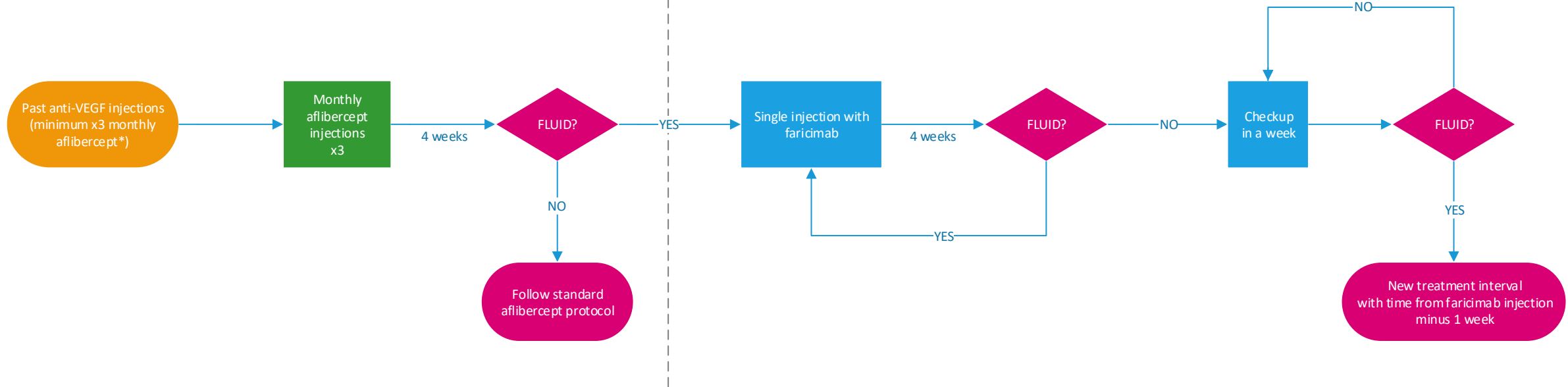

Supplement: Supplementary file 1 — Supplementary file1 (PDF 83 KB) [file 417_2024_6421_MOESM1_ESM.pdf]
