# Supplementary material for: Short-term outcomes of treatment switch to faricimab in patients with aflibercept-resistant neovascular age-related macular degeneration
Source: Graefes Arch Clin Exp Ophthalmol. 2024 Feb 28;262(7):2153–62. doi: 10.1007/s00417-024-06421-0 (PMC11222265; doi:10.1007/s00417-024-06421-0)
Supplement: Supplementary file 2 — Supplementary file2 (DOCX 12 KB) [file 417_2024_6421_MOESM2_ESM.docx]

**Supplementary figure legend**

Supplementary figure 1. Flowchart of inclusion, treatment switch to faricimab and follow-up.

*: Ranibizumab treatment was allowed.
